# Ffar4 regulates inflammatory oxylipin balance in the brain

**DOI:** 10.1002/alz.094966

**Published:** 2025-01-09

**Authors:** Katherine A Murphy, Brian A Harsch, Gregory C Shearer, Timothy D O'Connell

**Affiliations:** ^1^ University of Minnesota, Minneapolis, MN USA; ^2^ Pennsylvania State University, University Park, PA USA

## Abstract

**Background:**

Oxylipins are oxygenated fatty acid (FA) metabolites that are important mediators of inflammation. Neuroinflammation is a hallmark of Alzheimer’s disease (AD), and brains of AD patients contain more pro‐inflammatory and less anti‐inflammatory oxylipins compared to healthy controls. Free fatty acid receptor 4 (Ffar4) is a G‐protein coupled receptor for medium and long‐chain FAs, including, but not limited to, omega‐3‐polyunsaturated FAs. Ffar4 is expressed in a variety of tissues, including neurons, astrocytes, and microglia. Ffar4 signaling produces anti‐inflammatory and pro‐resolving oxylipins that can attenuate inflammation, reduce oxidative stress, and prevent apoptosis. This suggests that targeting Ffar4 signaling will preserve a more anti‐inflammatory/pro‐resolving oxylipin profile and potentially improve outcomes.

**Method:**

We accessed a biorepository that contains de‐identified electronic health records linked to patient genetic data for associations with Ffar4 polymorphisms and health reports. APP^SAA^ knock‐in (KI) mice (Jackson, strain #034711) were used to model AD. Brain regions from wild‐type (WT), APP^SAA^ KI, Ffar4 knockout (KO), and APP^SAA^ KI/Ffar4KO mice underwent LC/MS/MS analysis to detect oxylipins. RAW264.7 cells were treated with the Ffar4 agonist, TUG‐891, and subjected to LC/MS/MS to detect oxylipins.

**Result:**

In humans, we discovered that the Ffar4 inactivating polymorphism, R270H, was associated with an increased risk of AD and memory loss. We observed pronounced alterations in the oxylipin content of brain tissues, with a reduction in anti‐inflammatory oxylipins in Ffar4KO brains compared to WT. APP^SAA^ KI mice had an increase in pro‐inflammatory oxylipins that was exacerbated by loss of Ffar4. Ffar4 activation with a synthetic agonist in a macrophage cell line resulted in increased production of anti‐inflammatory oxylipins, while decreasing pro‐inflammatory oxylipins. These data indicate that Ffar4 mediates oxylipin production and Ffar4 activation promotes the production of anti‐inflammatory and pro‐resolving oxylipins.

**Conclusion:**

The anti‐inflammatory properties of Ffar4 make it an intriguing target for diseases that result from chronic inflammation, such as AD. Our data show that Ffar4 can modulate the inflammatory tissue environment by controlling oxylipin synthesis. Given that Ffar4 attenuates inflammation, activating Ffar4 could mitigate AD‐related neuroinflammation. By targeting Ffar4 it might be possible to redirect the immune response, alter oxylipin profiles, and improved prognosis.

